# Urine as a complementary specimen for RT-qPCR detection of Oropouche virus

**DOI:** 10.1007/s00705-026-06615-3

**Published:** 2026-04-13

**Authors:** Suwellen Sardinha Dias de Azevedo, Anna Clara Gregório Có, Eric Arrivabene Tavares, Lucas André Silva Bonela, Jéssica Graça Sant’Anna, Priscila Marinho, Jaqueline Pegoretti Goulart, Luciana Polaco Covre, Isabela Ribeiro Rodrigues, Daniel Claudio Oliveira Gomes, Edson Delatorre, Rodrigo Ribeiro-Rodrigues

**Affiliations:** 1 Laboratório Central de Saúde Pública do Estado do Espírito Santo, Secretaria de Saúde do Estado do Espírito Santo, Avenida Marechal Mascarenhas de Moraes, 2025, Bento Ferreira, ES 29050–625 Vitória, Brazil; 2 Núcleo de Doenças Infecciosas, Universidade Federal do Espírito Santo, Avenida Marechal Campos, 1468, Maruípe, ES 29.043–900 Vitória, Brazil; 3 Escola Superior de Ciências da Santa Casa de Misericórdia de Vitória, Avenida Nossa Senhora da Penha, 2190, Santa Luíza, ES 29045–402 Vitória, Brazil; 4 Laboratório de Genômica e Ecologia Viral, Centro de Ciências da Saúde, Universidade Federal do Espírito Santo, Av. Marechal Campos, 1468, Maruípe, Vitória, ES 29040–090 Brazil

## Abstract

Oropouche fever is an emerging arboviral disease in South America, for which reliable molecular diagnostic specimens throughout the course of infection are essential for surveillance and clinical management. This study aimed to compare the diagnostic performance of RT-qPCR in paired urine and serum samples and to evaluate temporal trends in cycle threshold (Ct) values according to time since symptom onset. A retrospective analysis was conducted on 41 paired serum–urine samples (one pair per patient) collected within 15 days after symptom onset. RT-qPCR results were classified as detectable or undetectable, with Ct values recorded when available. Positivity rates were compared using McNemar’s test, inter-matrix agreement was assessed with Cohen’s kappa, and paired Ct values were analyzed using the Wilcoxon signed-rank test. Temporal trends were evaluated according to clinical phases defined by time since symptom onset: acute phase (0–7 days) and early convalescent phase (8–14 days), and univariable logistic regression was used to assess the effect of time on detection probability. Urine samples showed a significantly higher positivity rate (75.6%, 31/41) than serum samples (43.9%, 18/41; McNemar *p* = 0.037), with poor agreement between matrices (Cohen’s kappa ≈ − 0.52). While serum Ct values increased over time), indicating declining detectability, urine Ct values remained showed no significant temporal trend throughout the two clinical phases. No significant association was observed between days since symptom onset and urine detection probability within 15 days. These findings suggest that urine may serve as a complementary specimen for molecular detection of Oropouche virus, particularly when serum testing occurs later in the course of infection.

## Introduction

Oropouche fever is a zoonotic arboviral disease caused by Oropouche virus (OROV), an orthobunyavirus belonging to the family *Peribunyaviridae* with a tripartite negative-sense RNA genome composed of three segments (L, M, and S). The virus is primarily transmitted by the midge *Culicoides paraensis*. First identified in Trinidad and Tobago in 1955, its circulation was historically confined to the Amazon region of South America, where transmission is sustained in sylvatic cycles [1]. Since 2022, however, OROV has expanded beyond these traditional endemic areas [2]. In 2024, Brazil experienced widespread outbreaks, with Espírito Santo emerging as a major hotspot—the state reporting the highest number of confirmed cases nationwide in both 2024 and 2025 [3].

Clinically, Oropouche fever presents as an acute febrile illness resembling dengue, chikungunya, or other arboviral infections. Prior to 2024, OROV infection was regarded as self-limited and non-lethal. However, following its spread to regions beyond the Amazon Basin, severe manifestations have been documented, including neurological complications [4], adverse pregnancy outcomes [5], and a small number of confirmed fatalities [6, 7]. Given the nonspecific symptoms at onset—fever, headache, myalgia, arthralgia, and rash—laboratory confirmation is essential [1]. Laboratory diagnosis of OROV infection relies primarily on molecular detection of viral RNA by RT-qPCR during the acute phase of illness. Serological assays, including IgM and IgG detection, may support diagnosis in later stages but are limited by potential cross-reactivity with other orthobunyaviruses and by delayed development of detectable antibody responses. As a result, molecular testing remains the preferred diagnostic approach during the early phase of infection. The current gold standard relies on RT-qPCR detection of viral RNA in serum or plasma, which is most sensitive within the first five days after symptom onset, when viremia peaks. Beyond this window, diagnostic sensitivity declines markedly.

Alternative clinical specimens, particularly urine, have shown diagnostic advantages for several RNA viruses transmitted by arthropod vectors. For Zika virus, RNA persists longer and at higher levels in urine than in serum—a pattern consistently observed during outbreaks in French Polynesia and Brazil, where urine also facilitated detection of asymptomatic cases [8, 9]. Urine collection is noninvasive, making it particularly advantageous for newborns and infants. Preliminary studies have suggested similar utility for OROV, with RNA detectable in urine beyond five days post-symptom onset [10, 11]. Collectively, these observations position urine as a promising surrogate or complementary specimen for OROV diagnosis, especially when serum collection is delayed or logistically difficult.

In this context, we investigated the diagnostic performance of urine during the ongoing OROV outbreak in Espírito Santo (2024–2025). Using paired serum and urine samples collected within 15 days of symptom onset, this study aimed to: (i) compare OROV RNA detection rates between the two matrices, and (ii) evaluate the temporal patterns of Ct values according to time since symptom onset. By analyzing paired specimens from the same individuals, we sought to assess whether urine may serve as a complementary clinical specimen for molecular detection of OROV, particularly when serum testing occurs later in the course of infection.

## Materials and methods

### Study design and setting

An observational diagnostic-comparison study was conducted in Espírito Santo, Brazil, from April 2024 to May 2025. Urine and serum specimens from symptomatic individuals across all 78 municipalities of the state were analyzed at the Central Public Health Laboratory (LACEN-ES). During the study period, 47,610 suspected arboviral cases were tested at LACEN-ES as part of the state surveillance system, of which 11,910 were confirmed as OROV-positive by RT-qPCR.

**Fig. 1:**
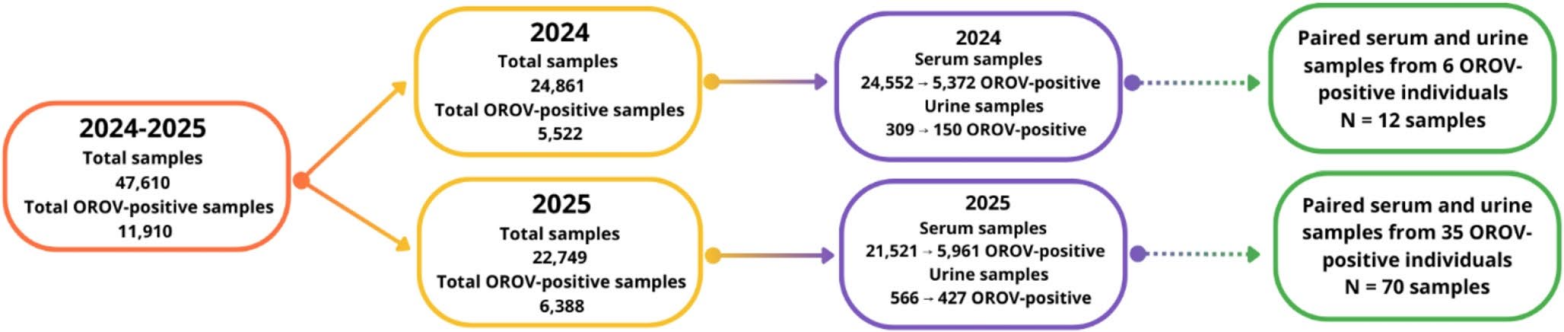
Study design and sample distribution (2024–2025). A total of 47,610 clinical samples were processed between April 2024 and May 2025, of which 11,910 tested positive for Oropouche virus (OROV). In 2024, 24,861 samples were analyzed, yielding 5,522 OROV-positive results (24,552 serum samples: 5,372 positive; 309 urine samples: 150 positive). In 2025, 22,749 samples were analyzed, with 6,388 OROV-positive results (21,521 serum samples tested: 5,961 positive; 566 urine samples tested: 427 positive). Only 41 OROV positive individuals presented paired serum and urine samples comprising a total of 82 samples (2024: 6 individuals, 12 paired samples; 2025: 35 individuals, 70 paired samples). This subset was used for comparative analyses of OROV RNA detection dynamics between serum and urine specimens

To explore the temporal patterns of OROV RNA detection at the population level, an exploratory analysis was conducted using a large dataset of independent urine and serum samples. This analysis was intended to generate hypotheses regarding differences in temporal Ct trajectories between specimen types and to complement the primary paired comparison. This non-paired dataset was generated by randomly selecting 530 urine and 535 serum samples from the pool of OROV-positive patients detected during the study period. Random selection was stratified to match the distribution of days since symptom onset, sex, and age across both specimen types. Following this exploratory phase, a subset of paired urine and serum samples was analyzed for confirmatory comparisons (“paired dataset”). Among the confirmed OROV-positive records, 41 paired specimens met the inclusion criteria: (i) both urine and serum samples obtained from the same patient; (ii) collection within 15 days of symptom onset; and (iii) at least one of the two specimens positive for OROV (Fig. 1). Serum and urine specimens were obtained either on the same day (50%) or within a short interval between collections (median difference = 1 day; IQR 0–3), reflecting routine diagnostic sampling in the surveillance system. Subsequent analyses using these paired samples followed the same analytical procedures described above.

### Sample processing and RT-qPCR detection

All specimens were processed according to LACEN-ES’ routine protocols for arboviruses diagnosis. OROV RNA was detected using the RT-qPCR described by Naveca et al. [12], which targets conserved regions of the S segment of OROV. Amplification reactions were interpreted according to the routine diagnostic criteria used at LACEN-ES. Samples were classified as detectable when amplification occurred with a cycle threshold (Ct) value below 40, and as undetectable when no amplification signal was observed or when Ct values exceeded this threshold. Ct values were recorded for all detectable reactions. Dates of specimen collection and symptom onset were obtained to calculate the interval (days) between onset and sampling. For temporal analyses, samples were categorized according to time since symptom onset into two clinical phases: acute phase (0–7 days) and early convalescent phase (8–14 days); comparisons were restricted to pairs collected within 15 days of symptom onset.

### Data sources and ethical considerations

Patient demographic (age and sex) and epidemiological data (municipality, symptoms, and date of symptom onset) were retrieved from LACEN-ES’s Laboratory Management System (GAL) and, for confirmed cases, cross-referenced with the Brazilian Ministry of Health’s eSUS system. All samples were anonymized prior to analysis. The study was approved by the Human Research Ethics Committee at the University of Vila Velha (CAAE no. 84698324.7.0000.5064), which waived written consent for use of de-identified diagnostic specimens.

### Statistical analysis

Two analytical approaches were employed: an exploratory analysis using non-paired samples and a comparative analysis using paired specimens. For the exploratory analysis, the non-paired dataset was used to fit a linear regression model adjusted for age and sex, including sample type (urine or serum), days since symptom onset, and their interaction as predictors. Model coefficients were examined to evaluate whether the temporal dynamics of Ct values differed between specimen types. For the exploratory analysis using independent urine and serum samples, Ct values were analyzed according to days since symptom onset. A linear regression model adjusted for age and sex was used to evaluate temporal trends in Ct values across the sampling interval.

For the comparative analysis, the primary outcome was the difference in OROV RNA detection rates between urine and serum in paired samples. Paired proportions were compared using McNemar’s test, and agreement between specimen types was quantified using Cohen’s kappa coefficient. For pairs with quantitative Ct values available for both specimen types, differences were assessed using Wilcoxon signed-rank test, to account for the paired structure of the data and the non-normal distribution of Ct values. Spearman’s rank correlation was used to evaluate the association between Ct values in paired specimens. Ct values were summarized as medians and interquartile ranges (IQRs) and compared across clinical phases using the Kruskal–Wallis test. The effect of days since symptom onset on OROV RNA detection probability was further explored using univariable logistic regression, with results expressed as odds ratios (ORs) and 95% confidence intervals (CIs). All analyses were conducted in R software version 4.5.1 [13], and a two-sided p-value < 0.05 was considered statistically significant.

## Results

### Exploratory analysis of non-paired samples suggests prolonged detectability of OROV RNA in urine

During the study period, 47,610 suspected arboviral cases were tested at LACEN-ES as part of the state surveillance system, of which 11,910 were confirmed as OROV-positive by RT-qPCR (Fig. 1). From this population, two analytical datasets were constructed: an exploratory dataset of independent urine and serum samples to assess temporal patterns of viral RNA detection, and a confirmatory dataset consisting of paired urine–serum specimens obtained from the same individuals.

**Fig. 2:**
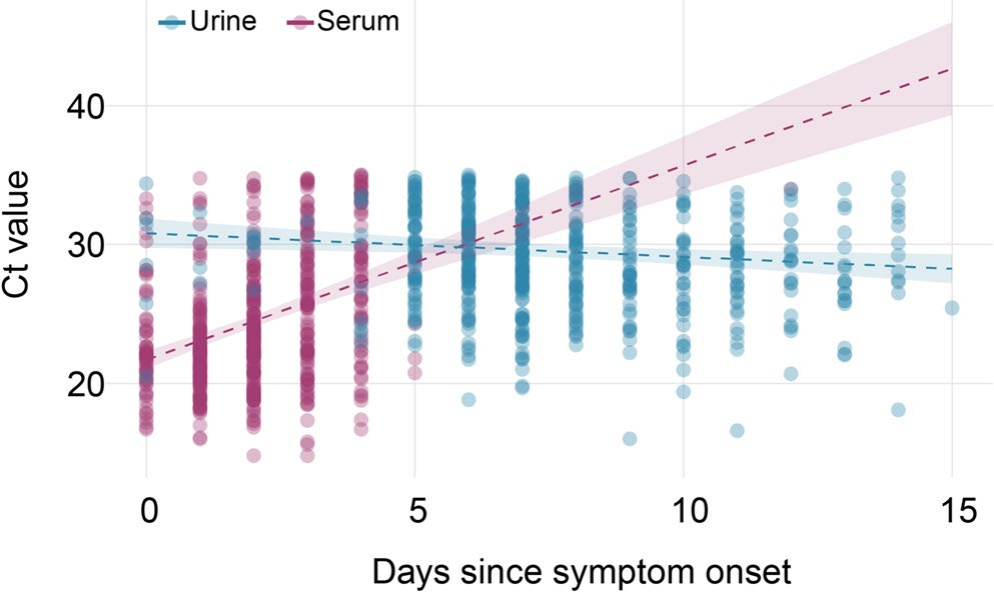
Temporal dynamics of Ct values in non-paired serum and urine samples for Oropouche virus (OROV) detection. Scatterplots illustrate changes in Ct values (inversely proportional to OROV RNA concentration) over days since sumptom onset. A linear model adjusted for age and sex showed a progressive decline in serum but stable values in urine, supporting prolonged detectability of OROV RNA in urine compared to serum

To explore temporal patterns of OROV RNA detection at the population level, Ct values were analyzed from 530 urine and 535 serum samples received at LACEN-ES during the study period. Serum samples showed a clear increase in Ct values with time (β = 1.40; *p* < 0.001), consistent with progressive decline in viral RNA detectability. In contrast, urine samples exhibited higher initial Ct values but remained relatively stable over the same period, as evidenced by a significant interaction between time and specimen type (β = −1.57; *p* < 0.001). These findings demonstrate that OROV RNA persists longer in urine than in serum, reinforcing the value of urine as a complementary specimen for molecular diagnosis – particularly in later stages of infection (Fig. 2).

### Demographic and clinical characteristics of patients with paired urine and serum samples

Among the 41 patients with paired serum and urine samples, OROV detection patterns were distributed as follows: 23 (56.1%) were positive only in urine, 10 (24.4%) only in serum, and 8 (19.5%) in both matrices. Comparative analyses revealed notable differences across these groups (Table 1).

**Table 1:** Characteristics of study participants stratified by OROV detection in serum and/or urine

Characteristic	OROV-positive serum and urine *n* = 8^1^	OROV-positive serum only *n* = 10^1^	OROV-positive urine only *n* = 23^1^	*p*-value^2^
Sex				0.068
Female	7 (88%)	10 (100%)	15 (65%)	
Male	1 (13%)	0 (0%)	8 (35%)	
Age (years)	38 (22)	27 (7)	49 (16)	0.002
Age group (years)				0.005
0–18	2 (25%)	1 (10%)	0 (0%)	
19–30	1 (13%)	6 (60%)	2 (8.7%)	
31–50	2 (25%)	3 (30%)	11 (48%)	
51–70	3 (38%)	0 (0%)	7 (30%)	
71+	0 (0%)	0 (0%)	3 (13%)	
Pregnancy				< 0.001
No	5 (63%)	1 (10%)	19 (83%)	
Yes	3 (38%)	9 (90%)	4 (17%)	
Hospitalization				0.047
No	5 (63%)	8 (80%)	22 (96%)	
Yes	3 (38%)	2 (20%)	1 (4.3%)	
Days Post-Symptom Onset - Urine	7.1 (2.6)	-	6.3 (3.4)	0.027
Days Post-Symptom Onset - Serum	5.88 (3.80)	3.80 (1.99)	-	

Notes: ^1^ n (%) / Mean (SD); ^2^ Fisher’s exact test; Kruskal-Wallis rank sum test

Patients with urine-only positivity were significantly older (mean age 49 years) than those with serum-only positivity (27 years) or positivity in both matrices (38 years; *p* = 0.002). Pregnancy status was strongly associated with detectable viremia: 90% of serum-only positive patients were pregnant, compared with 38% in the both-positive group and 17% in the urine-only group (*p* < 0.001). Hospitalization rates were higher in the both-positive group (38%) than in the urine-only group (4.3%; *p* = 0.047). The mean interval from symptom onset to sample collection was longer for urine in the both-positive group (7.1 days) than in the serum-only group (3.9 days; *p* = 0.027). No significant differences were observed in the prevalence of comorbidities.

Geographically, the 41 paired samples represented 26% (20/78) of municipalities in Espírito Santo. Three municipalities contributed the majority of pairs: Santa Teresa (seven pairs), Afonso Cláudio (six pairs), and São Roque do Canaã (four pairs).

### Diagnostic performance of urine versus serum for OROV detection

**Fig. 3:**
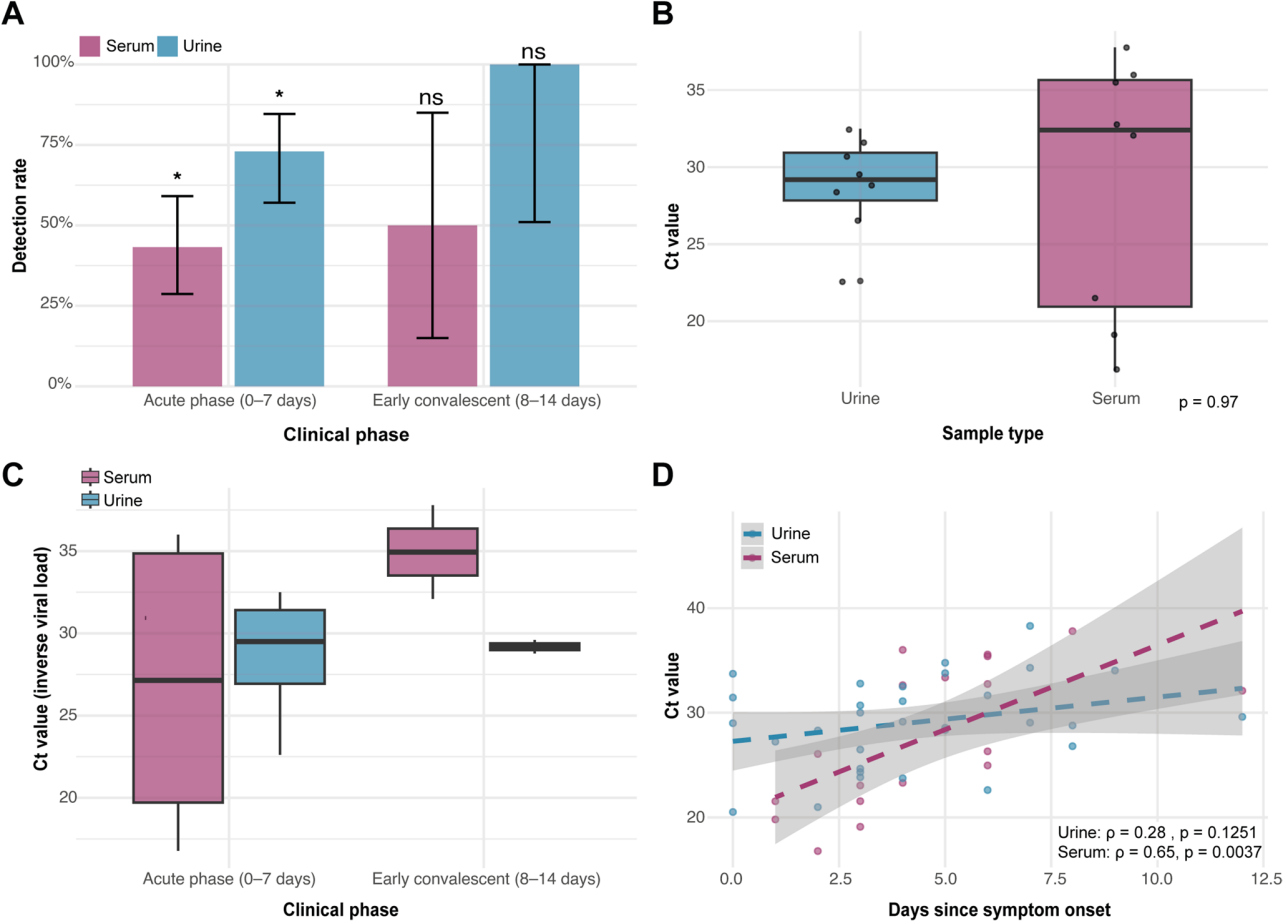
Diagnostic performance of Oropouche virus (OROV) in different sample types according to time since symptom onset. (**A**) Detection rate of OROV in serum and urine according to clinical phase (acute phase: 0–7 days; early convalescent phase: 8–14 days) (* *p* = 0.018, ns = not significant). (**B**) Ct values in paired urine and serum samples (Wilcoxon signed-rank test t, *p* = 0.97). (**C**) Comparison of Ct values between urine and serum stratified by clinical phase. No statistical differences were found. (**D**) Correlation between Ct values and days since symptom onset for urine (ρ = 0.28) and serum (ρ = 0.65). Lower Ct values correspond to higher viral load (inverse relationship)

Overall, urine samples demonstrated higher OROV RNA detection rate than serum (75.6% [31/41] vs. 43.9% [18/41], *p* = 0.007), a tendency that persisted across clinical phases (Fig. 3A). During the acute phase (0–7 days after symptom onset), OROV RNA was detected in 73.0% of urine samples compared with 43.2% of serum samples, representing a statistically significant difference between matrices (*p* = 0.018). In constrast, during the early convalescent phase (8–14 days), detection rates remained higher in urine (100%) than in serum (50%), although this difference was not statistically significant (*p* = 0.414), likely due to the small number of paired samples in this interval. McNemar’s test confirmed significant asymmetry in discordant pairs (*p* = 0.037), indicating that urine detected multiple cases missed by serum. Cohen’s kappa revealed poor agreement between the two matrices (κ ≈ −0.52), underscoring their lack of interchangeability. When serum was taken as the reference standard, urine exhibited a sensitivity of 44.4% (8/18), driven primarily by the high proportion of urine-positive/serum-negative results. Together, these findings indicate that urine can identify infections undetected in serum, highlighting its value as a complementary diagnostic specimen.

### Temporal dynamics of OROV Ct values in paired serum and urine samples

Among the subset of patients with paired Ct values available in both matrices (*n* = 8), there was no significant difference between urine and serum Ct values (paired t-test, *p* = 0.97), and the correlation between them was negligible (*r* ≈ 0.02) (Fig. 3B). Median Ct values were comparable (urine: 28.4 [IQR 26.7–30.5] vs. serum: 28.5 [IQR 27.2–31.0]), indicating similar viral loads when both matrices are positive. However, the absence of correlation suggests that viral burden in one matrix cannot be reliably inferred from the other.

When stratified by clinical phase, serum Ct values showed clear tendency to increase between the acute phase (0–7 days) and the early convalescent phase (8–14 days), consistent with declining viremia over time. In contrast, urine Ct values remained comparatively stable across phases (Fig. 3C). Pairwise comparisons between specimen types within each phase did not reveal statistically significantly differences. Nevertheless, the observed divergence in Ct trajectories suggests that viral RNA clearance occurs earlier in serum, whereas urine maintains detectable RNA levels later in the course of infection.

Regression analyses further confirmed these patterns: serum Ct values exhibited a moderate positive correlation with days since symptom onset (ρ = 0.65, *p* = 0.0037), reflecting progressive viral clearance, whereas urine Ct values showed only a weak, non-significant correlation (ρ = 0.28, *p* = 0.1251) (Fig. 3D). In multivariable logistic regression adjusted for age, sex, and pregnancy status, no covariates were significantly associated with serum positivity, including time since symptom onset (OR ≈ 1.00, *p* = 0.98). By contrast, urine positivity displayed non-significant trends toward higher odds with increasing days since symptom onset (OR ≈ 1.70 per day, *p* = 0.083) and older age (OR ≈ 1.21 per year, *p* = 0.051). Although these associations did not reach statistical significance, they align with the observation that urine positivity persists later in the disease course, reinforcing its potential to provide a broader diagnostic window than serum.

## Discussion

The data presented here support the concept that urine may represent a complementary specimen for the molecular detecction of OROV, particularly beyond the acute phase of infection. Regression analysis adjusted for age and sex consistently showed that serum OROV Ct values increased over time, reflecting viral clearance, whereas urine OROV Ct values remained relatively stable. The significant positive correlation between days since symptom onset and specimen type further confirms that RNA is detectable for a shorter period in serum and more consistently in urine. Urine samples yielded higher positivity rates than serum, maintaining RNA detection up to 15 days after symptom onset, while serum performance declined markedly after the acute phase of infection. These findings highlight the potential of urine as either a surrogate or complementary diagnostic matrix, particularly in settings where blood collection is delayed or infeasible, such as in children and neonates.

Our results are consistent with previous studies reporting OROV RNA detection in urine and saliva as alternative specimens [10, 11]. More recent longitudinal investigations have demonstrated prolonged OROV RNA persistence in urine and other body fluids, including semen and whole blood, compared with serum, with evidence of ongoing replication beyond the acute phase [14, 15]. The biological mechanisms underlying the presence of OROV RNA in urine and other body fluids remain incompletely understood. One possible explanation is renal filtration of circulating viral RNA or virions during the viremic phase, followed by delayed clearance from the urinary tract [14]. Alternatively, viral RNA detection in urine and genital secretions may reflect localized infection or persistence within urogenital tissues, as described for other arboviruses such as Zika virus [16, 17]. In such scenarios, viral RNA may persist in tissue or immune-privileged compartments even after clearance from the bloodstream. However, detection of viral RNA alone does not necessarily indicate the presence of replication-competent virus, and further studies integrating viral culture and longitudinal sampling are required to clarify these mechanisms [14]. Although whole blood may represent a sensitive specimen for viral detection, its routine use in clinical diagnostics may be limited by technical and operational challenges related to collection, processing, and storage. These findings suggest that viral RNA may persist longer in cellular compartments than in serum, supporting the investigation of alternative clinical matrices for OROV diagnosis. Collectively, these observations strengthen the hypothesis that urine sampling may extend the diagnostic window for OROV detection, warranting its incorporation into diagnostic algorithms and surveillance strategies.

Our results align with published observations for other arboviruses, in which urine has extended the diagnostic window compared with serum or plasma [10, 11]. However, the exploratory analysis based on non-paired samples should be interpreted cautiously, as it was not designed to directly compare specimen types within individuals. Nevertheless, the patterns observed were consistent with those identified in the paired analysis, supporting the hypothesis that OROV RNA may persist longer in urine than in serum. During the ZIKV epidemic, urine became a preferred specimen in many settings due to its prolonged detectability and higher viral loads, thereby improving diagnostic sensitivity and facilitating surveillance of asymptomatic infections [8, 17]. Similar patterns have been reported for DENV and WNV, where viral RNA was detectable in urine after clearance from serum, supporting its utility for late confirmation of infection [18, 19]. For CHIKV, evidence is less consistent, with some studies showing limited added value of urine beyond the acute phase [20]. Within this broader context, our findings for OROV reinforce the emerging consensus that urine is a valuable diagnostic matrix for arboviruses, particularly for extending detection beyond the first week of illness [10, 11, 14]. By providing paired analyses in a well-characterized outbreak cohort, our study contributes robust evidence to this growing literature.

We also observed associations between detection patterns and patient characteristics. Urine-only positivity was more common in older patients, whereas serum-only detection was associated with pregnancy. These trends may reflect age-related immune changes [21] and pregnancy-induced immune adaptations [22] influencing viral kinetics and shedding. Although limited by sample size, these findings suggest that host immune status may shape detection profiles. The higher hospitalization rate among patients positive in both matrices suggests a potential link between systemic dissemination and disease severity, though this requires further investigation.

Analysis of Ct values further highlighted the distinct temporal patterns between the two specimen types. Serum Ct values increased progressively with time since symptom onset, consistent with declining detectability as viremia resolves. In contrast, urine Ct values showed no significant temporal trend within the observation window. Although these findings should not be interpreted as evidence of longitudinal persistnce, they suggest that viral RNA may remain detectable in urine across a broader time interval than in serum when samples are collected at different stages of infection. Multivariable models identified no significant predictors of serum positivity, whereasurine positivity showed non-significant trends toward association with older age and longer time since symptom onset. Although these associations did not reach statistical significance, they align with recent longitudinal data showing prolonged OROV RNA persistence in urine and other body fluids—sometimes for weeks after the acute phase [14, 15]. The biological mechanisms underlying this pattern remain incompletely understood. Future studies employing simultaneous viral culture and genomic characterization are needed to distinguish non-infectious RNA persistence from ongoing replication with potential transmission implications [14].

Our study has several limitations that must be considered. First, the small sample size constrained statistical power, a common challenge in studies of emerging arboviruses [23]. Second, the absence of a control group with paired negative samples limited robust specificity estimates, as seen in early evaluations of other arboviruses [17, 20]. Third, viral load was semi-quantitatively assessed via Ct values rather than absolute quantification, hindering direct cross-study comparisons [24]. Finally, we could not determine whether urine RNA represented an intact infectious virus or merely genomic fragments, as virus isolation was not performed—an uncertainty noted in prior reports [15, 18]. These limitations highlight opportunities for future refinement but do not detract from the internal consistency of our findings or their implications.

In summary, urine sampling may extend the diagnostic window for OROV detection and complement serum-based molecular diagnosis. A key contribution of this study is demonstrating that urine sampling extends the diagnostic window up to two weeks after symptom onset. Incorporating urine into diagnostic algorithms could enhance case confirmation and surveillance sensitivity, especially in resource-limited settings or when serum collection is delayed or logistically challenging. Larger cohorts with longitudinal follow-up are warranted to validate these findings, elucidate mechanisms of RNA persistence, and evaluate implications for transmission dynamics and clinical management.

## Data Availability

The datasets generated and/or analysed during the current study contain sensitive patient information and, therefore, are not publicly available. Access may be granted upon reasonable request to the corresponding author, subject to approval by the relevant ethics committee.
